# Early detection of language categories in face perception

**DOI:** 10.1038/s41598-021-89007-8

**Published:** 2021-05-06

**Authors:** Cristina Baus, Elisa Ruiz-Tada, Carles Escera, Albert Costa

**Affiliations:** 1 Department of Cognition, Development and Educational Psychology, University of Barcelona, 08035 Barcelona, Spain; 2 Center for Brain and Cognition, CBC, Pompeu Fabra University, Barcelona, Spain; 3 University of Tokyo, Tokyo, Japan; 4 Brainlab-Cognitive Neuroscience Research Group, Department of Clinical Psychology and Psychobiology, University of Barcelona, Barcelona, Spain; 5 Institute of Neurosciences, University of Barcelona, Barcelona, Spain; 6 Institut de Recerca Sant Joan de Déu, Esplugues de Llobregat, Barcelona, Spain

**Keywords:** Psychology, Human behaviour

## Abstract

Does language categorization influence face identification? The present study addressed this question by means of two experiments. First, to establish language categorization of faces, the memory confusion paradigm was used to create two language categories of faces, Spanish and English. Subsequently, participants underwent an oddball paradigm, in which faces that had been previously paired with one of the two languages (Spanish or English), were presented. We measured EEG perceptual differences (vMMN) between standard and two types of deviant faces: within-language category (faces sharing language with standards) or between-language category (faces paired with the other language). Participants were more likely to confuse faces within the language category than between categories, an index that faces were categorized by language. At the neural level, early vMMN were obtained for between-language category faces, but not for within-language category faces. At a later stage, however, larger vMMNs were obtained for those faces from the same language category. Our results showed that language is a relevant social cue that individuals used to categorize others and this categorization subsequently affects face perception.

## Introduction

The ability to quickly extract socially relevant information from others is of great importance in our daily social interactions. Faces play a major role in identifying, categorizing and recognizing others. Information about person’s identity (e.g., gender), ethnicity, emotional states (e.g., anger) or even personality traits (e.g., trustworthiness), is readily and rapidly extracted from a face and used as a categorization cue shaping person identification (e.g.^1–3^). ERP studies have been very relevant in showing that categorical information of socially relevant cues is automatically codified during face perception (e.g., facial emotions; e.g.^4–6^). Indeed, modulations of face-sensitive ERP components (e.g., N170) to social category information (race, sex) has been taken as evidence that mechanisms of social categorization operate in parallel with those underlying structural encoding of a face (e.g.^7,8^).

While the sensitivity of perceivers to social category information is evident for cues directly obtained from the face, such as race, age or gender, little is known about other sources of category information that although relevant for interpersonal interaction are not directly conveyed in a face. One of those is language, considered a dimension of social categorization^9^ that provides important information not only about language content (what is being said) but also about the speakers’ identity (who is saying it^10^). Considering the relevance of language in social interaction, the question we address in this study is whether information about the speaker’s language, native or foreign, is automatically obtained and use to categorize others’ faces.

Perhaps the most direct evidence that face and language interact comes from research on bilingualism showing that the face of an interlocutor serves as a cue that determines bilinguals’ language use^11–16^. In particular, the study of Martin et al.^13^ showed that the ease with which a language can be predicted is detected early after presenting a face. In an EEG experiment, faces for which the speaking language could be easily predicted (i.e., faces previously speaking only one of the bilingual’s two languages) elicited a larger early negativity than those for which language could not be predicted (faces mixing the bilingual’s two languages during familiarization). Those results are an indication that faces might convey information pertaining to the language of the speaker.

Expanding upon these findings, the present study investigated whether bilingual perceivers automatically categorize individuals’ faces depending on the language they speak (either the bilingual’s native or foreign language). To do so, we drew from oddball studies identifying the vMMN (visual mismatch negativity) in response to top-down effects of language knowledge on categorical perception^17–19^. Several studies have showed that language-specific terminology affects the perception of colors (e.g.^19–20^), shapes (e.g.^21^), and objects (e.g.^22,23^). This effect, known as the “Whorfian effect” in visual perception, has been often interpreted as a result of language tuning perception to category-relevant stimulus features in a top-down fashion^24–26^, but see^27^.

Relevant here, Yu et al.^21^ revealed early modulations of the vMMN associated with newly learned categories of faces. Participants learned to assign faces to two different artificially-created linguistic categories and then underwent a visual oddball paradigm with infrequent faces (deviant) presented among frequent faces (standard). The deviancy effect (vMMN) was compared for faces previously learned as belonging to the same category of the standard (deviant within-category faces) or belonging to a different category (deviant between-category faces). Early vMMN (N170) and late vMMN (P200) were elicited for between-language categories but not for within-category ones, revealing the rapid effect of learned categories on face perception. In the present study, we followed a similar experimental procedure as in Yu et al.^21^ to explore the effect of language categorization during face perception.

### The present study

Two experiments were conducted. The first experiment was created to induce the language categories of faces for the participants, and the second to explore the effect of language categorization on the electrophysiology of face perception.

By means of the Memory Confusion Paradigm (MCP, also known as “Who said what”^28,29^, we first examined whether faces were categorized by language (Spanish or English). The MCP is a well-established approach of measuring implicit social categorization^30,31^. The reasoning behind the utilization of this paradigm is as follows: if a particular feature of a person—such as the language they speak—is a basis for categorization, then people who share those characteristics are more easily confused among each other during recognition (i.e., Spanish-paired faces will be confused with other Spanish-paired faces, while English-paired faces will be confused with other English-paired faces). This can occur without the participant’s conscious awareness of this happening and therefore reveals fundamental, implicit and automatic categorization processes.

Once language categorization was established, an oddball paradigm was used to test differences in face perception between standard faces and within-language category deviant faces (e.g., a Spanish face presented among Spanish-paired faces as standards) and between-language category deviant faces (e.g., a Spanish face occurring among English-paired faces as standards).

## Methods

### Participants

A total of 57 (29 males; mean age = 21.4, SD = 2.3) participants took part in the study from the database at Pompeu Fabra University who were Spanish dominant speakers and had English as a foreign language. Speech comprehension in English was evaluated by asking participants to listen to a 6 min recording and then respond to seven different comprehension questions. Participants responded correctly an average of 5.4 (SD = 1.6), showing that they were mid-proficiency English listeners. Due to technical and/or artifact rejection, data from five participants were not included in the final analysis.

All of the participants were selected from the participants’ database of the Neuroscience laboratory and signed a consent form before starting the experiment. All methods were in accordance with the guidelines of the ethics committee at the University Pompeu Fabra and approval of the experimental protocol was obtained from the University ethical committee (CEIC-Comité Étic d’Investigació Clínica).

### Stimuli

Grey-scale photos of eight Caucasian males with neutral expressions were selected from the AR face database^33^. They all had dark hair and dark eyes, so that they would be convincing as both a Spanish and/or English speakers, and none of them had any easily distinguishable facial characteristics. The average dimension of the faces employed was 4.7 cm width (SD = 0.18) and 6.8 cm height (SD = 0.28).

Twenty-four neutral, non-autobiographical sentences were created in Spanish, as well as the English translation of each (e.g., *El libro tiene cien páginas* for “The book has a hundred pages”). On average, Spanish phrases were 4.9 words in length (range 4–7 words), while English phrases (range 4–7 words) were 5.1 words in length. No statistical difference was found between sentence lengths of the two languages (t(23) = −1.072, *p* = 0.3). Phrases were recorded and edited using Audacity (v 2.0.3) from four native male Spanish speakers for Spanish sentences, and four native male English speakers for English sentences.

### Procedure

**Figure 1:**
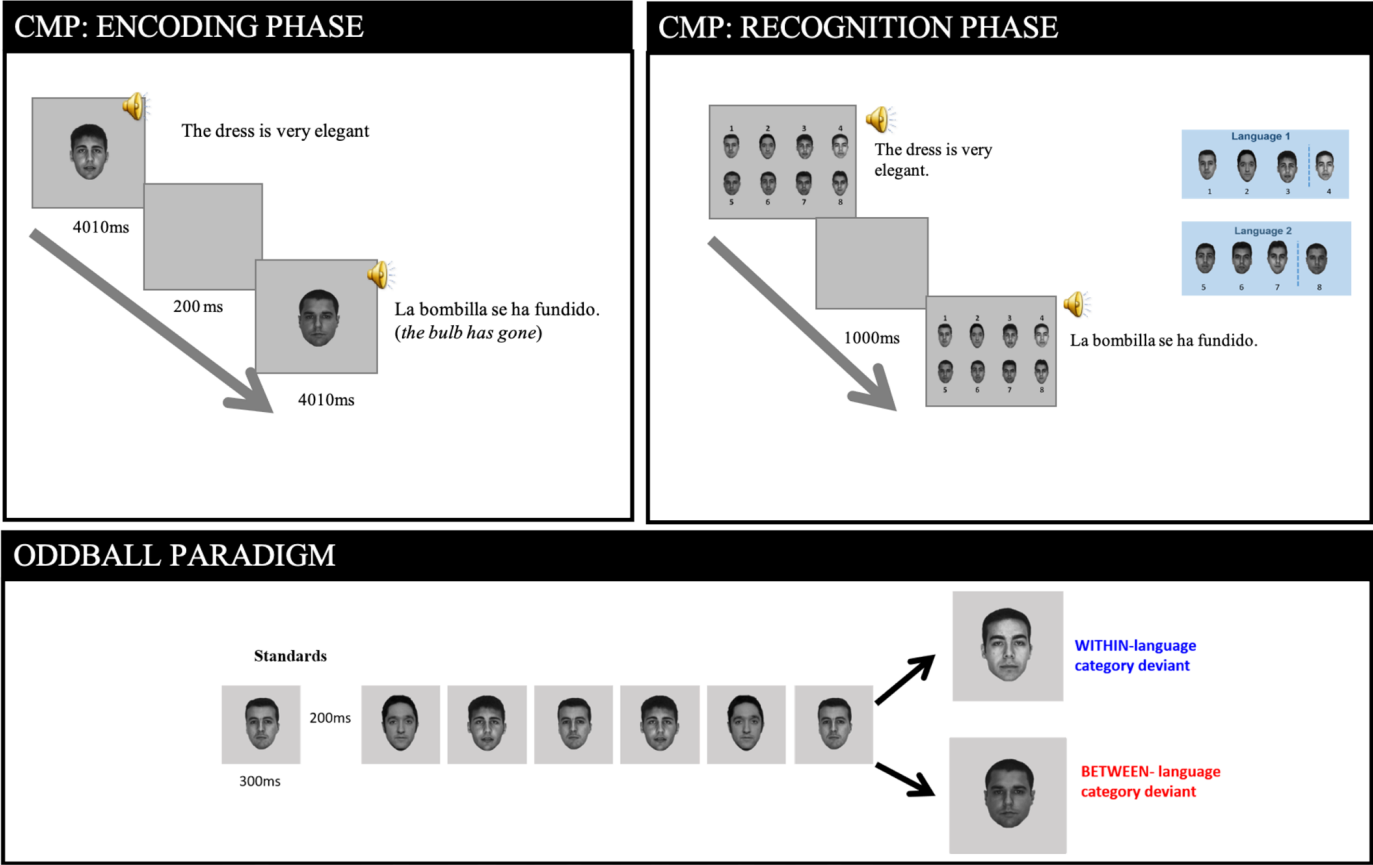
Illustration of the experimental paradigm. Upper panel represents de Confusion Memory Paradigm. Lower panel represents de Oddball Paradigm. The oddball sequence represents a block in which faces 1–4 in the CMP have been paired to one language and 5–8 to the other language.

Participants were seated in front of a computer screen (at 60 cm distance approximately) in an EEG cabin after instructions were given and consent forms were signed. The experiment was presented on a TFM 20” monitor (refresh rate 60 Hz) and controlled using the software E-prime 2.0^34^. Figure 1 illustrates the experimental design comprising the Memory Confusion Paradigm and the Oddball paradigm.

#### Memory confusion paradigm

The MCP consisted of two main phases, the Encoding and the Recognition phase. During the Encoding phase, individual photos were presented on the screen while phrases were auditorily presented through headphones. Participants were instructed to form impressions of the speakers while they watched and listened, as they would be asked questions later regarding this phase of the experiment.

Each of the eight faces appeared three times during the Encoding phase, for a total of 24 presentations. The three presentations of each face contained different sentences, but voices were kept the same. That is, each face always had the same voice, but the information provided in each of the three sentences was different. Eighteen lists were created to counterbalance across face, sentence and language. Therefore, all faces were accompanied by every sentence in both languages across participants.

Photo and audio were presented simultaneously, with the photo remaining on screen for a total of 4010 ms. This display time was selected to allow the face to appear an extra 2000 ms after the longest sentence had ended (which was 2010 ms in length), allowing the face to be encoded in depth. A blank grey screen appeared for 200 ms between each face.

During the Recognition phase, all eight photos were presented in one screen (two rows, four columns), numbered 1–8. Eight different face templates were created so that each of the eight faces appeared in a different position across templates. Face templates were counterbalanced across participants but the face frame was the same across trials for a given participant (i.e., faces were on the same exact position for a given participant).

A trial started with the presentation of a face template including the eight faces. After 200 ms, while the face template remained on the screen, one of the auditory sentences were presented (randomly selected). Participants had to decide which of the eight faces was accompanied by that specific sentence in the Encoding phase (i.e., “to which face the voice belongs”) by pressing the corresponding number on the keyboard. The face template remained on screen until the participant responded. After the response, there was a blank screen for 1000 ms. The procedure was the same for all 24 sentences that the participants had heard in the Encoding phase were presented.

After the EEG experiment (see below), a second recognition test was administered with the objective of checking whether participants had retained the language categories throughout the oddball test. The procedure was exactly the same as the first Recognition test except that it was not preceded by an Encoding phase.

#### Visual oddball paradigm

Faces that had previously been categorized as Spanish or English speakers were shown to the participants in a fast and consecutive manner, where some faces were repeated so that they occurred frequently (i.e., standard faces) and other faces occurred less frequently as deviant faces. Importantly, depending on the language with which a face was paired in the MCP and the sequence of faces in which it was embedded, deviants could be within-language category or between-language category.

Participants completed eight blocks, each with 844 face presentations, including 748 standards, 48 deviant within-language category, and 48 deviant between-language category. Four blocks had Spanish-paired faces as the standard condition and the other four had English-paired faces as the standards. Thus, the final experiment included 5984 standard trials, 384 deviant-within language category trials and 384 deviant-between language category trials.

A reverse control procedure was used. Each of the eight faces appeared in all three conditions (standard, deviant within-language category and deviant between-language category), counterbalanced across blocks.

Within a block, three of the four faces from one language group were repeated as standard faces and the deviant condition was either the fourth face from the same language group (deviant from within a language category) or a deviant from the other language group (deviant from between language categories). Within deviants, forty-eight of those were from the same language category as the standard (deviant within-language category), as well as 12 presentations for each of the four faces from the other language category (deviant between-language category). Deviants were presented after seven, eight or nine standards (equally distributed), with 31 sequences per block where a deviant did not appear.

To prevent the number of deviant tokens from possibly confounding the results (i.e., a given between-language deviant face was presented less frequently that a given within-language deviant face), in a subsample of participants (n = 25), the experimental design carried out was the same but with the alteration that within and between-language deviants were presented with the same frequency. That is, instead of presenting each of the four between-language faces 12 times, we presented one of the between-language faces 48 times (as done for the within-language condition). In doing so, we eliminated the possibility that any difference between deviants was due to differences in the frequency of presentation of the two deviant types.

Standard and deviant faces were presented on the center of the screen for 300 ms with an ISI between faces of 200 ms. Framed faces were presented until the participant responded, or for a maximum of 1000 ms, in order to allow participants to blink. To ensure that participants were attending to the task, 48 faces (from the standard condition) were used as catch trials within each block. Faces were framed by an outlined box and participants were instructed to press a button when face were presented. Neither those faces nor the sequence of standards/deviants in which they were embedded were considered in the analysis.

#### Behavioral analysis

In the MCP, participant errors were collected and analyzed. Errors were differentiated as within-language category errors or between-language category errors. To correct for the discrepancy in the chances of making a within-language category error (3/4) versus a between-language category error (4/4), between-language error rate was multiplied by 0.75.

#### EEG recording and analysis

EEG was recorded using Brain Vision (BrainProducts GmbH, Gilching, Germany) with 32 active electrodes (ActiCap) mounted according to the Standard International 10–20 system and referenced to the left mastoid. The sampling rate was 500 Hz. The impedance was kept under 15 kΩ. Horizontal EOG was recorded from electrodes attached to both the left and right outer canthi of the eyes, and vertical movement was monitored with an electrode placed below the right eye. ERP analysis was carried out using MATLAB (R2010b version 7.11.0.584) and the EEGlab Toolbox^35^. The data went through an offline band-pass filter of 0.08–25 Hz and was re-referenced to the average activity of the two mastoids. ICA was implemented (FastICA implemented in EEGlab) and eye movement components were removed. Bad electrodes were interpolated from surrounding electrodes. Then, ERP waveforms of the different trials were averaged per participant, with an epoch from − 100 to 450 ms, and baseline corrected. Artifacts were rejected when surpassing a window threshold of − 75 to 75 µV. Cleaned ERPs were time locked to the onset of every face presentation (standards, within-language category deviants and between-language category deviants). For the analyses, only standards immediately preceding the deviants were considered. However, given that the number of trials included in the standard condition was double than those included in the two deviant conditions, a random sample of 275 standard trials was included in the final analysis. Thus, the final analysis included on average 265 standard trials (range 218–275), 249 deviant within-language category trials (range 203–274), and 244 trials (range 187–269).

The analyses focused on the vMMN within-language category and between-language category. The two vMMN responses for the two language categories were calculated as the difference between the standard (random sample of trials) and each of the deviant conditions. Mean amplitudes of the two vMMN conditions were compared within two time-windows: the first, matching the N170 latency (vMMN within the 130–200 ms time-window) and the second, matching the P200 latency (P200^36^, vMMN within the 200–300 time-window). The vMMN within the N170 time-window was selected following the maximum peak of the N170 (around 170 ms after the onset presentation of a visual stimulus), which is especially evident for faces at posterior (e.g., P7/P8) electrode clusters and has been taken as an index of the neural mechanisms underlying change detection and pre-attentional processes^37^, for a review, see^38^. Previous face perception findings have revealed differences between vMMN effects of within-category and between-category deviants at the N170 latency (e.g.^21^).

The time-window for the vMMN within the N170 range was centered on the maximal peak latency at posterior electrodes (155 ms) and included neighboring time points (130–200 ms). The P200 is less described in the vMMN literature and it is still debated whether this component is indicative of either pre-attentive processes, post-perceptual processing^39^ or an attentional shift^36^. In the color perception literature, modulations of the vMMN within the P200 latency have been revealed for color categories trained in the laboratory^39^, a finding that holds relevance for the present study. The time-window for the late vMMN within the P200 was centered on maximal positive peak latency across electrodes (246 ms) and included neighboring time points as well (200–300 ms).

Mean amplitude analyses of the vMMN within the N170 time-window focused on electrodes within the posterior region. A 2 × 2 repeated measures ANOVA including deviancy (vMMN within-language category and vMMN between-language category) and Laterality (left and right). The two posterior regions included the average activity of four electrodes (Posterior Left: CP5, P7, P3, O1; Posterior Right: CP6, P4, P8, O2).

Mean amplitude analyses of the vMMN within the P200 time-window were conducted in a 2 × 2 × 2 ANOVA including deviancy (vMMN within-language category and vMMN between-language category), region (fronto-central and posterior), and Laterality (left and right) as main factors. The average activity of four electrodes comprised each region of interest (Fronto-central Left: F3, FC1, FC5, C3; Fronto-central Right: F4, FC2, FC6, C4 Posterior Left: CP5, P7, P3, O1; Posterior Right: CP6, P4, P8, O2).

## Results

### Behavioral results

In the Recognition test, participants made an average of 18.5 total errors (SD = 2.8) out of 24 responses (75% error rate). As predicted, participants made significantly more within-language category errors (10.2, SD = 2.7) than between-language category errors (6.2, SD = 2.7, t(51) = 5.62, *p* < 0.001; *d* = 1.48). This indicates that participants did in fact categorize faces according to the language they were accompanied with. This result was further validated in the second Recognition test carried out after the oddball paradigm (t(51) = 5.1, *p* < 0.001, *d* = 1.3). An ANOVA comparing performance in the two Recognition tests revealed no significant differences between the two tests (F < 1), which shows that language categorization effects remained present through the entire experimental session.

### Event-related potentials

**Figure 2:**
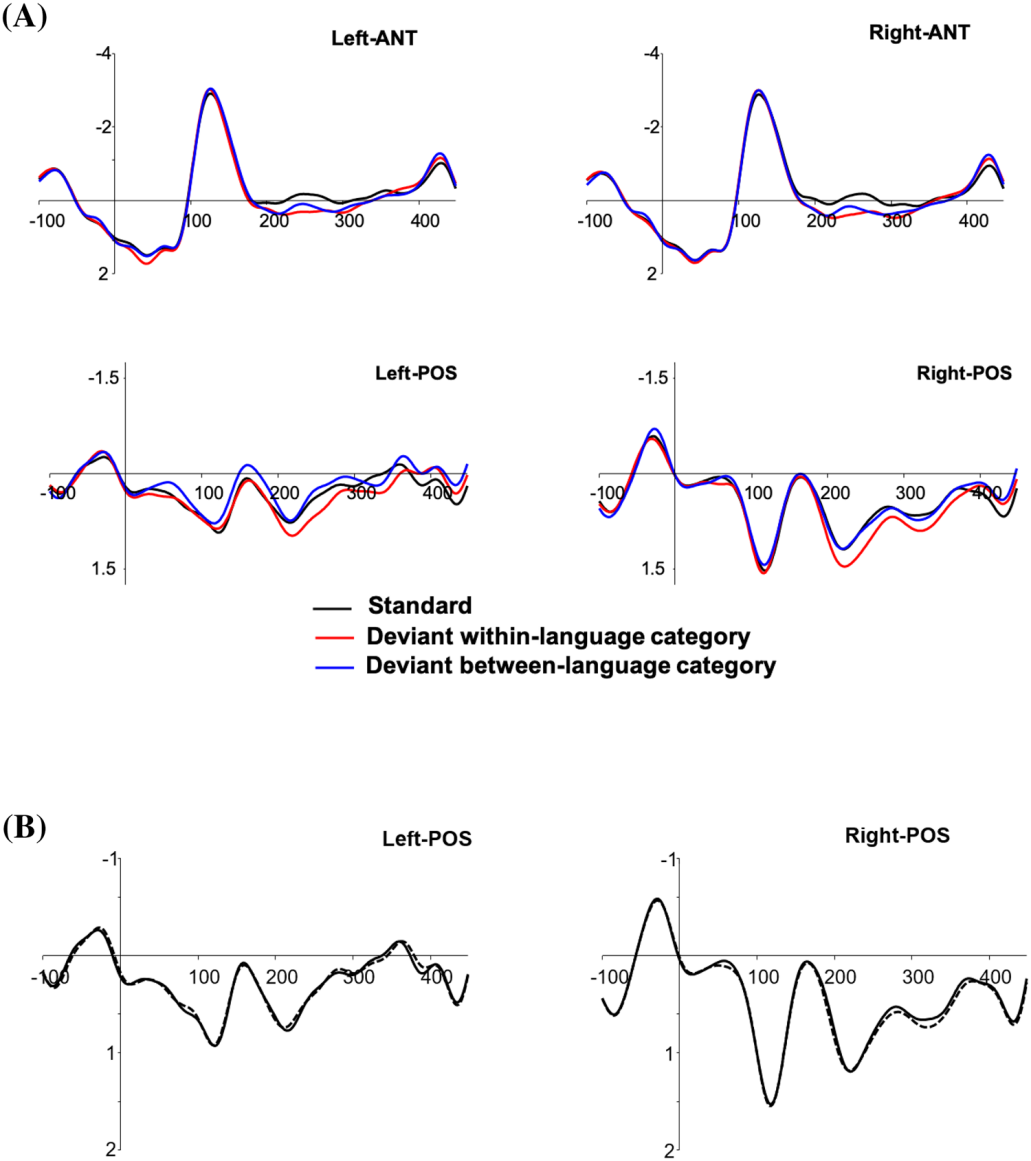
(**A**) Waveforms of the grand-averages of the three conditions (ERPlab Toolbox^40^). Each figure represents the linear derivation of four electrodes within the region of interest. Negative is plotted up. *ANT* anterior electrode clusters, *POS* posterior electrode clusters. (**B**) Waveforms of the grand-averages of the two standard conditions. Each figure represents the linear derivation of four electrodes within the region of interest. Negative is plotted up. *POS* posterior electrode clusters.

Figure 2A represents waveforms of the standard (random sample) and the two deviant conditions. Figure 2B represents ERP waveforms of the standard condition considering all trials (dashed line) and the standard condition considering the random sample of trials (solid line).

The vMMNs for the within and between-language categories were calculated by subtracting the mean amplitude of the deviant ERP minus the mean amplitude of the standard ERP. This was calculated for the two time-windows of interest (N170: 130–200 ms; P200: 200–300 ms).

### vMMN within the N170 time-window

**Figure 3:**
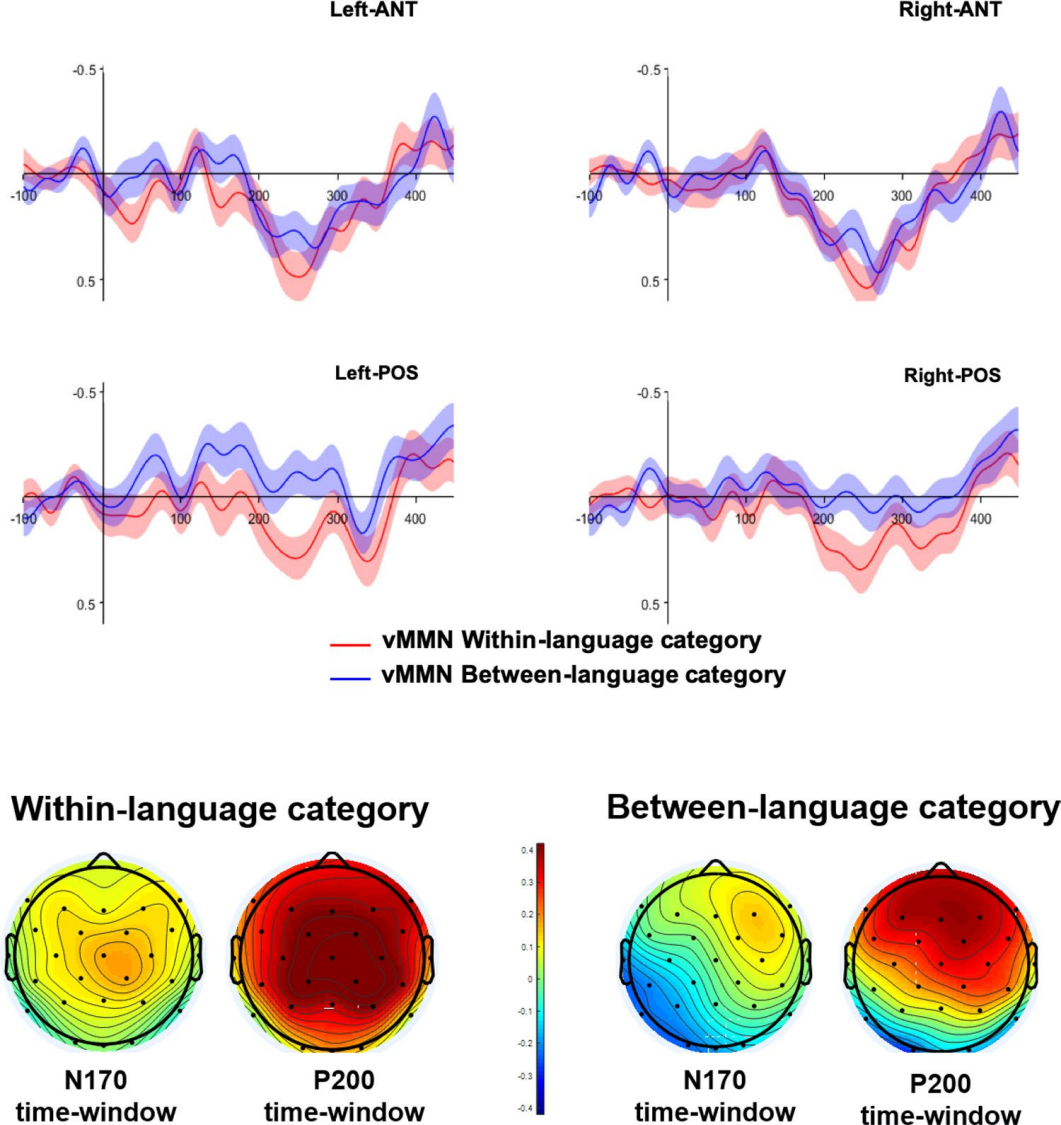
Upper panel: vMMN responses for within-language category paired-faces (red line) and between-language category paired-aces (blue line). Negative is plotted up. Shaded lines represent the mean standard error. Lower panel: topographies of the vMMN (deviant minus standard waveforms) at the N170 and P200 time-ranges (ERPlab Toolbox^40^).

**Figure 4:**
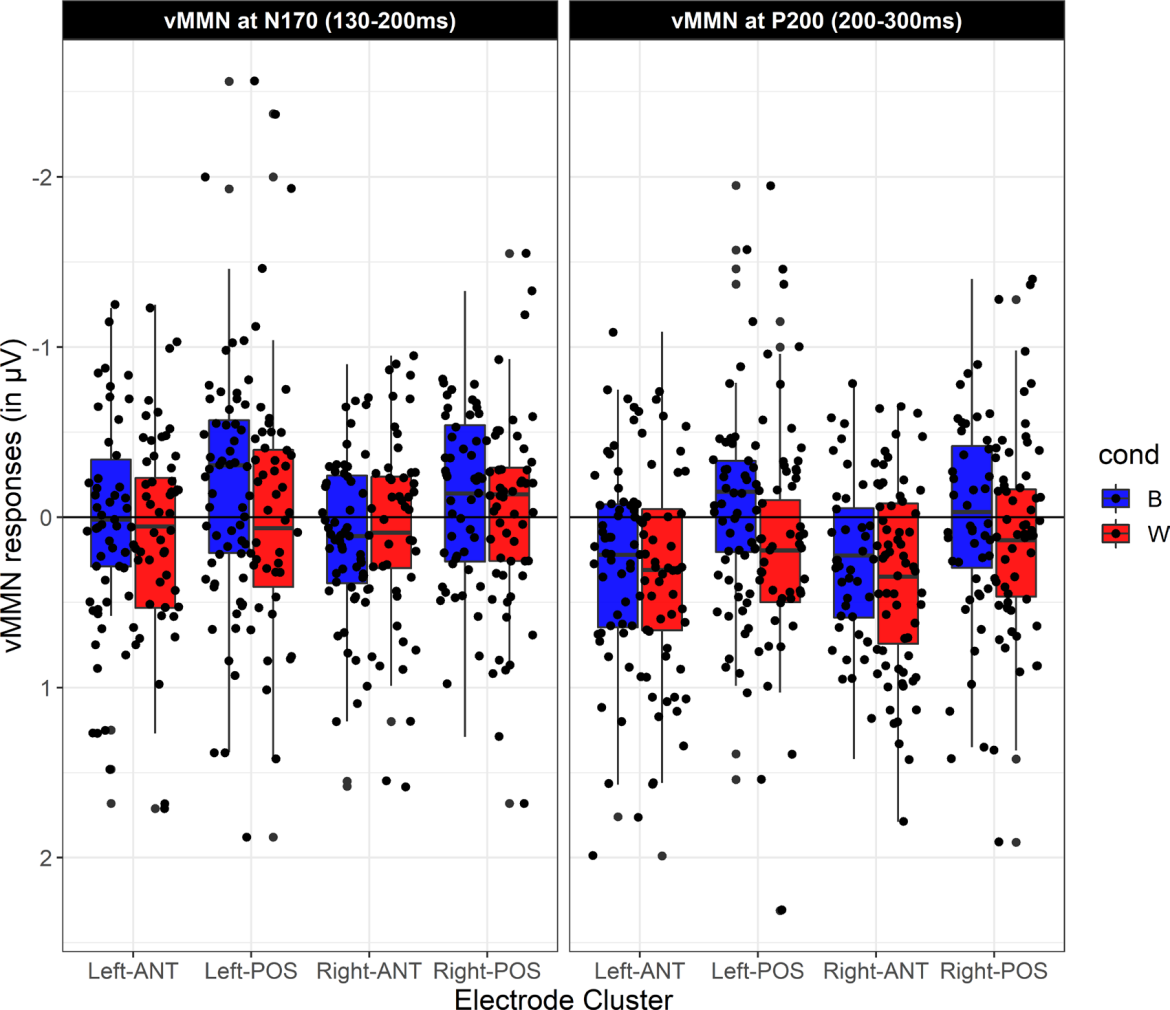
Boxplot of the vMMN for within (red) and between-language categories (blue) across regions of interest and time-windows (ggplot2 commands on R-Studio^41^. Negative is plotted up.

For the vMMN within the N170 time-window, the 2 × 2 repeated-measures ANOVA was conducted over posterior electrodes with the factors vMMN (within vs between) and lateratity (left vs right). The results revealed a main effect of condition, with larger vMMN for between-language category faces compared to within-language category faces (F(1,51) = 7.6, *p* < 0.05, η_p_^2^ = 0.13). Deviancy interacted significantly with laterality (F(1,51) = 7.5, *p* < 0.05, η_p_^2^ = 0.12), revealing that the vMMN for between-language category faces was greater than the vMMN within-language category faces within the left cluster of electrodes (F(1,51) = 12.8, *p* < 0.001, η_p_^2^ = 0.20), but not within the right cluster of electrodes (F(1,51) = 1.4, *p* = 0.2, η_p_^2^ = 0.02) (see Figs. 3 and 4). (A second analysis considered whether the vMMN was modulated by the version of the Experiment (version 1, where the frequency of deviants was different and version 2, where the frequency of deviants was the same). For the vMMN within the N170 time-window, the results revealed only main effect of condition (F(1,50) = 7.49, *p* = 0.009, η_p_^2^ = 0.13), an interaction with laterality (F(1,50) = 7.46, *p* = 0.009, η_p_^2^ = 0.13). Version of the experiment did not show neither a main effect (F(1,50) = 1.8, *p* = 0.17, η_p_^2^ = 0.03), nor an interaction with condition (F < 1) or condition and laterality (F < 1). The effect of condition was very similar in the two version of the experiment (Version 1: − 0.20 µV, Version2: − 0.24 µV). For the vMMN within the N170, the version of the Experiment did not interact with condition (F(1,50) = 1.1, *p* = 0.28, η_p_^2^ = 0.02) or with condition and region (F(1,50) = 3.4, *p* = 0.28, η_p_^2^ = 0.06). The main effect of Experiment was not significant (F(1,50) = 1.5, *p* = 0.22, η_p_^2^ = 0.03), revealing a very similar vMMN in the two version of the Experiment (Version 1: − 0.22 µV, Version2: − 0.23 µV).

### vMMN within the P200 time-window

For the vMMN within the P200 time-window, a 2 × 2 × 2 repeated-measures ANOVA was conducted with the factors deviancy (within vs between), laterality (left vs right) and region (anterior vs posterior). The results revealed a main a greater positivity for vMMN within-language category faces than for between-language category faces (F(1,51) = 8.07, *p* < 0.05, η_p_^2^ = 0.13). Deviancy significantly interacted with region (F(1,51) = 9.7, *p* < 0.05, η_p_^2^ = 0.16). The two vMMN only differed significantly within the posterior electrode cluster (F(1,51) = 16.1, *p* < 0.001, η_p_^2^ = 0.24) but not within the anterior one (F(1,51) = 1.3, *p* = 0.2, η_p_^2^ = 0.02) (see Figs. 3 and 4). Any other interaction resulted significant.

**Figure 5:**
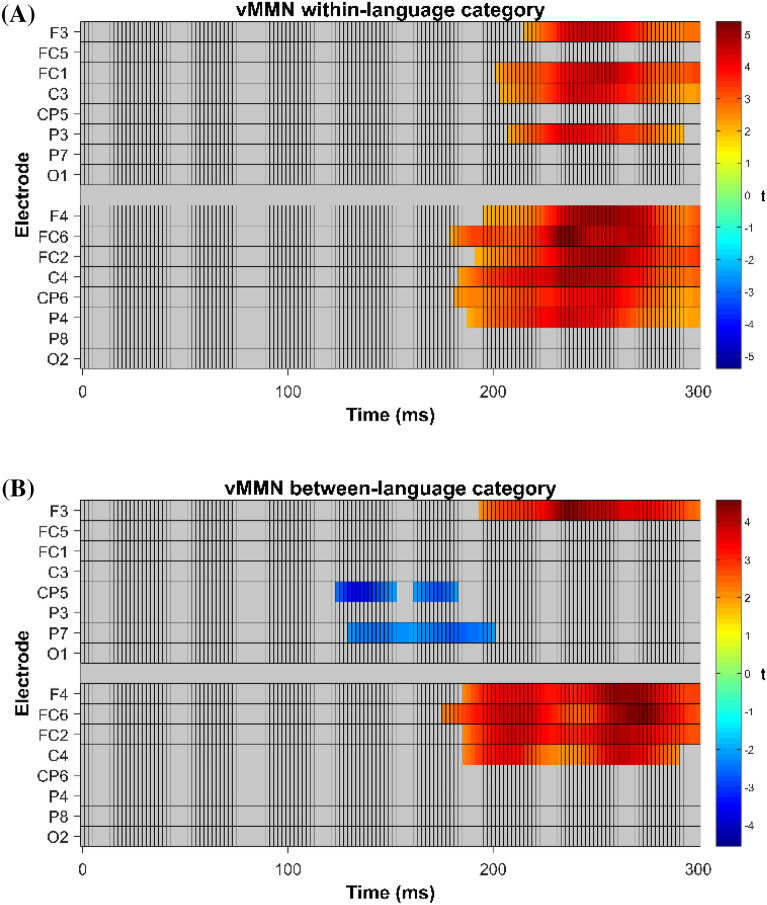
Cluster based permutation t-test^42^ on selected electrodes and time-points for vMMN within-category faces (**A**) and vMMN between-category faces (**B**). Alpha level of significance 0.05, number of permutations 2500.

These results were further validated in a cluster-based permutation t-test (^42^; Fig. 5) across time (0–300 ms) and selected electrodes for the within and between-language category vMMN (significant differences from zero). Cluster-based permutation test for the vMMN within-language category showed a positive cluster (positive-going vMMN) starting at 180 ms after the onset of the face presentation for electrode FC6, following then for multiple electrodes. Cluster-based permutation test for the vMMN between-language category showed two significant clusters: a negative significant cluster over posterior left electrodes CP5 and P7(starting at 124 ms for CP5) followed by a positive significant cluster over anterior electrodes (starting at 176 ms for FC6 and followed then by several anterior electrodes).

In summary, these results indicate that language categorization influences early stages during face processing, with an earlier detection of changes when deviant faces crossed the language category. Following this early detection, effects were larger for faces within the same language-category than for those crossing the language category.

## Discussion

In the present study, we tested top-down influences of language categorization on face processing. In a series of behavioral and EEG experiments, we examined whether language is used as a social cue to classify faces, and whether this language categorization affects early perception of faces.

Within the memory confusion paradigm (MCP), participants were more likely to confuse faces from within the same language group than from the other language group. That is, faces that were originally presented with Spanish phrases were more likely to be confused with other Spanish-paired faces than with English-paired faces, and English-paired faces were more likely to be confused with other English-paired faces than with Spanish-paired faces. The MCP relies on the type of errors made (within-category and between-category) to determine whether social categories have been created^32,30,31^. In particular, the observation that more within than between-language errors were committed suggests that language is used as a social cue to categorize other individuals’ faces. Interestingly, this categorization effect was present over an hour after participants’ initial exposure to the language categories. Finding the same effects in both Recognition phases (before and after the oddball paradigm) indicates that participants maintained the categories throughout the entire EEG experiment and this had an effect on face processing.

Indeed, we reported a two-step vMMN response in categorical face perception by language. Modulations in the vMMN within the N170 time-window and within the P200 time-window, with maximum strength at left-lateralized posterior electrodes, were sensitive to the language categories created, suggesting the involvement of automatic detection processes and attention towards the language with which faces were familiarized.

Within the time-range of the N170, the vMMN was elicited by faces crossing the language category but not for those belonging to the same language category as the standard ones. This result expands upon previous results reporting vMMNs associated with pre-attentive and automatic detection of changes (e.g.^18,43,44^) in perceptual categories of socially relevant face stimuli such as emotion, gender or race (e.g.^45,46^). Importantly, the effect of language categories was obtained when faces could not be sorted based on any other visual feature, suggesting that language is an important social cue that we automatically use to categorize others (e.g.^47^). The reported early vMMN also favors the Whorfian hypothesis of language knowledge affecting visual categorization^24,25^ of colors (e.g.^18^), objects^22^, word-meaning^48^ and visual speech perception (Files et al. 2013).

Language categorization was also reflected in the P200 range, with two interesting results arising. First, a fronto-central general deviancy effect, in that similar vMMN for between and within-language category. Second, a larger vMMN was observed for within-language deviants than for between-language deviants at posterior sites. Therefore, this late time-window appeared to be sensitive to both deviancy detection as well as language categorization effects.

Modulations of the vMMN within the P200 latency in posterior electrodes have been previously reported in studies testing the effects of newly learned categories based on color, shape or face perception^21,39,49^, with vMMNs appearing for between-category stimuli and not within-category stimuli. These results clearly contrast with the ones reported here. Interestingly, the fact that we obtained posterior vMMNs for between-language category faces in the early time-window and for within-language category faces in the late vMMN might be an indication that early detection of faces crossing the language category was followed by a shift in attention to those faces belonging to the same language category (see^50^, for a similar interpretation in processing emotion in faces).

Differences between frontal and posterior regions have been previously explained for the P300 component, with the frontal P300 (P3a) showing top-down attention changes towards rare stimuli (e.g.^51–53^) and the posterior P300 (P3b) reflecting an update of representations in working memory once a stimulus has been categorized (e.g.^54^). Although our effects were observed at an earlier timing than the one suggested for the P300 (approximately 350–500 ms), they nicely fit with explanations of the P3a (frontal) reflecting deviancy effects and the P3b (posterior) reflecting language category-specific effects. In the context of face perception, modulations occurring around 250 ms after face onset presentation (sometimes showing inverse polarity; N200) have been associated to the deeper and more individuated processing of in-group members (e.g., race^55,56^. In line with this observation, deviant faces belonging to the same language category as the standard one could be processed in more detailed than those belonging to the other language category. While interesting, this is one possible explanation of our results. Further studies should investigate underlying psychological processes, especially post-perceptual effects of language categorization. Our main contribution here was to show that language shapes face processing, supporting models of face-language interactions at all levels during person perception^57^.

## Conclusion

Our current study shows that faces are categorized using the language they are accompanied by. We thereby add language as another dimension of social categories that have been tested before, including race, gender, age, university-affiliation^58^, socio-economic class^59^, accents^31^, or coalitional groups^30^. Additionally, our results show that language categorization affects both pre- and post-perceptual stages of face processing, supporting the increasing body of literature claiming that categories affect early visual perception in a top-down manner.
